# Prevalence of institutional delivery and its correlates amongst women of reproductive age in Mozambique: a cross-sectional analysis

**DOI:** 10.1186/s12978-020-0905-4

**Published:** 2020-04-16

**Authors:** Sanni Yaya, Dina Idriss-Wheeler, Gebretsadik Shibre, Agbessi Amouzou, Ghose Bishwajit

**Affiliations:** 1 School of International Development and Global Studies, Faculty of Social Sciences, University of Ottawa, 120 University Private, Ottawa, ON K1N 6N5 Canada; 2 The George Institute for Global Health, University of Oxford, Oxford, UK; 3 Faculty of Health Sciences, University of Ottawa, Ottawa, Canada; 4 Bloomberg School of Public Health, Johns Hopkins University, Baltimore, MD USA; 5 Department of Reproductive Health and Health Services Management, School of Public Health, Addis Ababa University, Addis Ababa, Ethiopia

**Keywords:** C-section, Facility delivery, Mozambique, Global health, Women’s health, demographic and health surveys

## Abstract

**Background:**

The healthcare system in Mozambique is striving to reduce the high maternal and child mortality rates and stay on par with the Sustainable Development Goals (SDG 3.1). A key strategy to curb maternal and child mortality is to promote the use of professional childbirth services proven to be highly effective in averting maternal deaths. Currently, little is known about the use of childbirth services in Mozambique. The present study investigated the prevalence of professional healthcare delivery services and identified their sociodemographic correlates.

**Methods:**

This study used cross-sectional data on 7080 women aged 15–49 years who reported having a child during the past 5 years. The data were collected from the 2011Mozambique Demographic and Health Survey. The outcome variables were the choice of childbirth services that included 1) place of delivery (respondent’s home versus health facility), and mode of delivery (caesarean section versus vaginal birth). Data were analyzed using descriptive and multivariate regression methods.

**Results:**

The prevalence of health facility and C-section delivery was 70.7 and 5.6%, respectively. There was a difference in the use of professional birthing services between urban and rural areas. Having better educational status and living in households of higher wealth quintiles showed a positive association with the use of facility delivery services among both urban and rural residents. Regarding ethnicity, women of Portugais [2.688,1.540,4.692], Cindau [1.876,1.423,2.474] and Xichangana [1.557,1.215,1.996] had relatively higher odds of using facility delivery services than others. Antenatal care (ANC) visits were a significant predictor of facility delivery services both in urban [OR = 1.655, 95%CI = 1.235,2.218] and rural [OR = 1.265, 95%CI = 1.108,1.445] areas. Among rural women, ANC visit was a significant predictor of C-section delivery [1.570,1.042,2.365].

**Conclusion:**

More than a quarter of the women in Mozambique were not using health facility delivery services, with the prevalence being noticeably lower in the rural areas.

## Plain English summary

The Republic of Mozambique is one of the least developed countries in Southern Africa with a large portion of the population living below the poverty line. Characterized by high fertility rates and plagued by high maternal and child mortality rates, preventative maternity services are underutilized or may be inaccessible. Approximately 30% of births in the region are attended by family members while 23–40% are attended by insufficiently trained traditional birth attendants. Access to skilled birth assistance and health facility delivery services requires financial resources and many women in Mozambique cannot afford necessary expensive procedures and services.

Currently, little is known about the use of childbirth services in Mozambique. Using the well-established Andersen’s Behavioural Model of Health Service, the present study investigated maternal healthcare service utilization and identified correlated sociodemographic variables. The study used the 2011 Mozambique Demographic and Health Survey cross-sectional data on 7080 women aged 15–49 years who reported having a child during the past 5 yrs.

Findings revealed more than a quarter of women in Mozambique were not using health facility delivery services, particularly in the rural areas. Women with higher educational status and those living in more affluent households were more likely to use health facility delivery services in both rural and urban regions. Furthermore, antenatal care (ANC) visits were a predictor of C-section delivery in rural regions.

## Background

The Republic of Mozambique is located in Southern Africa and is one of the least developed countries in the region [1, 2]. Since the end of the prolonged civil war in 1992 (1977–1992), the country has introduced a series of macroeconomic reforms to revitalize the economy and initiatives to improve the living standards of the population [3]. Despite the noticeable progress made in the areas of poverty reduction, a large proportion of the population continues to live below the poverty line and have significant challenges in securing basic amenities such as ensuring food security and accessing healthcare [4]. Higher fertility rates (5.24 birth per woman as of 2016), a predominantly rural distribution of the population (67.49 as of 2016), a relatively young age structure (45.2% under age 15), low life expectancy (59.31 years as of 2017), and high maternal and child mortality rates characterize the demography of Mozambique [5].

According to available estimates, the under-five mortality rate was 72 (per 1000 live births as of 2017) and that of maternal mortality was 480 (per 100,000 live births as of 2015) [1], down from 1500 in 2000; one of the highest rates globally [6]. Approximately 30% of births in sub-Saharan Africa are unattended or only attended by family members while about 23–40% are attended by traditional birth attendants (TBAs) [7]. Every day, globally, approximately 830 women die from pregnancy and childbirth-related complications [8]. These deaths, almost all of which take place in low-income countries, could have been averted through the use of quality obstetric services [9, 10]. In addition to the lives lost and the emotional distress caused by maternal or neonatal bereavement [11], there is a strong human rights component to maternal child mortality (MCM) which is shaping women’s reproductive health policy making mechanisms [12, 13].

Reproductive health services in Mozambique are inadequate to meet the needs of a growing population. This is particularly the case for services such as the availability of skilled birth assistance as well as equipment for providing sophisticated procedures such as C-sections [14, 15] which are expensive and lead to a significant financial burden for the mother and her family, especially in low-income settings like Mozambique where health insurance coverage is very low [16]. Preferences and utilization of healthcare services are inherently subjective and multifaced, and can themselves be influenced by a host of demographic, sociocultural, environmental, and economic determinants [17–21]. From previous studies, healthcare utilization can be conceptualized as an outcome of proximal and distal factors and their interplay shapes people’s perception of health and motivations for action. Understanding these determinants are necessary for making concrete and evidence-driven policy approaches to tackle maternal mortality in Mozambique. We undertook the present study to analyze open-access and nationally-representative data from Mozambique Demographic and Health Survey (DHS 2011). These findings will help advance the understanding of the sociodemographic inequalities in the uptake of skilled birth attendants (SBAs) and C-section services in Mozambique as well as in the neighbouring countries with similar economic and sociocultural environments.

## Methodology

### Data source

Data for this study were collected from the sixth round of Mozambique Demographic and Health Survey. The survey was conducted by the National Statistical Institute (Instituto Nacional de Estatística) and the Ministry of Health (MISAU). The work was finally supported by United States Agency for International Development of the United America (USAID) with Inner City Fund (ICF) International providing technical assistance. Sample population included eligible men (15–54 years) and women (15–49 years) residing in households in urban and rural areas, excluding institutions such as hospitals, hotels, dorms. Data collection was done through direct interviews using a tablet-type computer (Computer-Assisted Personal Interview) system and this process lasted from June 2011 to November 2011. Sampling was done using multistage cluster technique which involves stratifying the provinces into primary sampling units (PSUs), and then selecting of households each PSUs. Of the 13,964 households initially selected, a total of 13,718 women were finally interviewed, resulting in a 99% response rate. These details are available from the final report of Mozambique 2011 DHS and available here: https://dhsprogram.com/publications/publication-FR266-DHS-Final-Reports.cfm.

### Outcome measures

The outcome variables of interest were: 1) place of delivery: home versus health facility, 2) use of c-section: yes versus no.

### Explanatory variables

The selection of explanatory variables was guided by Andersen’s Behavioural Model of Health Service utilization which postulates that healthcare utilization is a function of three major factors: 1) predisposing factors, 2) enabling factors and 3) need factors [22]. For this study, the data were secondary and hence the selection of the explanatory variables in line with the behavioral model was not completely possible. Based on the availability in the dataset, the following are included in the analysis: Age (15–19, 20–24, 25–29, 30–34, 35–39, 40–44, 45–49); Residency (Urban, Rural); Education (No Education, Primary, Secondary, Higher); Husband’s education (No Education, Primary, Secondary, Higher); Occupation (Not Working, Professional/Technical/Managerial, Agricultural - Self Employed); Wealth quintile (Poorest, Poorer, Middle, Richer, Richest); Electronic Media Access (No, Yes); Heard of Family Planning (FP) on the internet (No, Yes); Religion (Islam, Other); Ethnicity (Emakhuwa, Portugais, Xichangana, Cisena, Elomwe, Cindau, Xitswa, Other); Parity (1–5, > 5); Sex of Household Head (Male, Female); Last Child Wanted (Wanted Then, Wanted No More); Place of Delivery (Home, Health facility).

### Data analysis

Data were analyzed with Stata version 14. Dataset was cleaned by applying the inclusion criteria: experience of at least 1 childbirth in the preceding 5 years. As the surveys used cluster sampling techniques, all analyses were adjusted for this by using the *svy* command [23]. This command uses the information on sampling weight, strata, and primary sampling unit provided with the datasets. Sample characteristics were described as frequencies and percentages. Prevalence of using facility delivery and C-section (for total, urban and rural sample) was presented as bar charts. The predictors of facility delivery and C-section were measured using multivariate analysis. As both of the variables were dichotomous, we used binary logistic regression models and the results expressed using odds ratios (OR) with 95% confidence intervals (CIs). Each of the outcome variables was analyzed separately for the pooled, urban and rural participants. Model fit statistics were run after the regression analyses using the variance inflation factor (VIF) command. No multi-collinearity was detected as VIF values were below 10 for all the models. All tests were two-tailed and were considered significant at alpha value of 5%.

## Results

### Sample description

The basic characteristics of the sample population were shown in Table 1. A greater proportion of the participants: were aged 20–29 years (48.98%), from rural residences (63.16%), had primary education (51.50%), had no employment (53.59%), from households with highest wealth quintile (25.92%), had access to electronic media (71.69%), were followers of Islam (71.12%), were of Xichanga ethnicity (19.66%), had 1–5 children (81.78%), were from male-headed households (64.90%), wanted the last child (79.86%), and received at least four ANC visits (70.75%).

**Table 1:** Sample characteristics (*n* = 7080)

	Freq.	Percent
**Age**		
15–19	813	11.48
20–24	1771	25.01
25–29	1697	23.97
30–34	1319	18.63
35–39	935	13.21
40–44	394	5.56
45–49	151	2.13
**Residency**	Freq.	Percent
Urban	2608	36.84
Rural	4472	63.16
**Education**	Freq.	Percent
No Education	2139	30.21
Primary	3646	51.50
Secondary	1,22	17.23
Higher	75	1.06
**Husband’s education**	Freq.	Percent
No Education	1731	26.36
Primary	3272	49.82
Secondary	1422	21.65
Higher	143	2.18
**Occupation**	Freq.	Percent
Not Working	3794	53.59
Professional/Technical/Managerial	1269	17.92
Agricultural - Self Employed	2017	28.49
**Wealth index**	Freq.	Percent
Poorest	1067	15.07
Poorer	1194	16.86
Middle	1328	18.76
Richer	1656	23.39
Richest	1835	25.92
**Electronic Media access**	Freq.	Percent
No	2004	28.31
Yes	5076	71.69
**Religion**	Freq.	Percent
Islam	5035	71.12
Other	2045	28.88
**Ethnicity**	Freq.	Percent
Emakhuwa	1241	17.53
Portugais	607	8.57
Xichangana	1392	19.66
Cisena	676	9.55
Elomwe	266	3.76
Cindau	426	6.02
Xitswa	368	5.20
Other	2104	29.72
**Parity**	Freq.	Percent
1–5	5,79	81.78
> 5	1,29	18.22
**Sex of household head**	Freq.	Percent
Male	4595	64.90
Female	2485	35.00
**Last child wanted**	Freq.	Percent
Wanted Then	5654	79.86
Wanted No More	1426	20.14
**ANC visits**	Freq.	Percent
< 4	2071	29.25
4 or more	5009	70.75

**Fig. 1:**
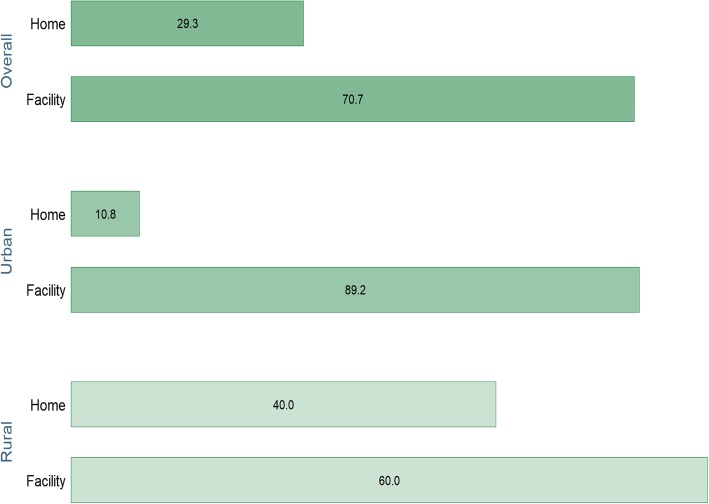
Prevalence of health facility delivery

Figure 1 shows that over three-fifth (70.7%) of the participants had their last childbirth at a health facility and 29.3% in their home. The percentage of home delivery was four times as high in the rural areas compared with urban areas (*p* < 0.05).

**Fig. 2:**
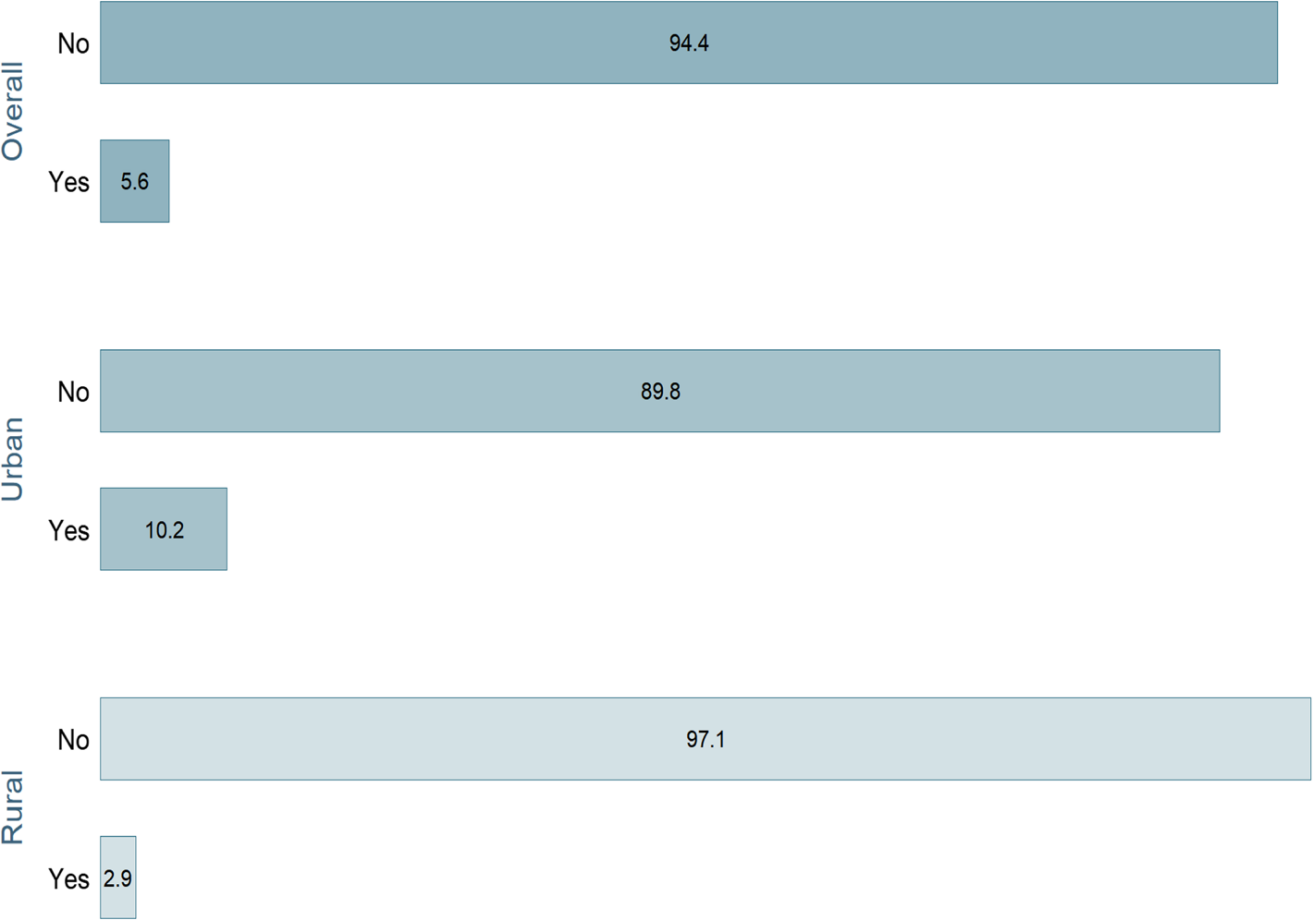
Prevalence of C-section delivery

Figure 2 shows that only 5.6% of the participants had their last childbirth using c-section. Similar to health facility delivery, the percentage of C-sections performed was markedly higher in the urban areas (10.2%) compared with rural areas (2.9%).

The predictors of using facility delivery and c-sections were presented in Table 2 and Table 3, respectively. In general, the odds of using facility delivery was lower among women in higher age groups. However, the difference was significant among those aged 40–44 years and in rural areas only. Rural residents had significantly lower odds of using facility delivery [OR = 0.527, 95%CI = 0.440,0.630]. Having primary, secondary, and higher education showed a positive association with the use of facility delivery services among both urban and rural residents. In the pooled sample, the odds of using facility delivery service were: primary [OR = 1.737, 95%CI = 1.370,2.203], secondary [OR = 2.608, 95%CI = 1.840,3.697], and higher [OR = 1.266, 1.012,1.584].

**Table 2:** Predictors of using Facility Delivery Services in Mozambique

	Pooled	Urban	Rural
**Age (15–19)**	1	1	1
20–24	0.888 [0.704,1.120]	0.726 [0.411,1.281]	0.918 [0.710,1.186]
25–29	0.815 [0.645,1.030]	0.826 [0.463,1.473]	0.796 [0.614,1.031]
30–34	0.869 [0.678,1.115]	1.020 [0.542,1.922]	0.834 [0.634,1.096]
35–39	0.830 [0.631,1.093]	0.696 [0.359,1.352]	0.853 [0.629,1.157]
40–44	0.688^*^ [0.493,0.959]	0.671 [0.305,1.477]	0.685^*^ [0.473,0.992]
45–49	0.725 [0.472,1.115]	0.380 [0.130,1.114]	0.820 [0.513,1.311]
**Residency (Urban)**	1		
Rural	0.527^***^ [0.440,0.630]		
**Education (No Education)**	1	1	1
Primary	1.190^*^ [1.039,1.363]	1.163 [0.791,1.710]	1.160^*^ [1.002,1.343]
Secondary	2.608^***^ [1.840,3.697]	1.860^*^ [1.030,3.358]	3.475^***^ [2.133,5.662]
Higher	1.266^*^ [1.012,1.584]	1.643^*^ [1.123,2.403]	1.251 [0.896,1.748]
**Husband’s education (No Education)**	1	1	1
Primary	0.979 [0.852,1.124]	0.953 [0.652,1.394]	0.980 [0.843,1.139]
Secondary	1.737^***^ [1.370,2.203]	1.616 [1.000,2.613]	1.762^***^ [1.328,2.338]
Higher	4.106 [0.553,30.47]	3.842 [0.497,29.68]	1.236^*^ [1.031,1.481]
**Employment (Not Working)**	1	1	1
Professional/Technical/Managerial	1.246^*^ [1.003,1.546]	1.189 [0.815,1.734]	1.302 [0.996,1.703]
Agricultural - Self Employed	0.998 [0.872,1.142]	0.778 [0.534,1.134]	1.060 [0.916,1.227]
**Wealth quintile (Poorest)**	1	1	1
Poorer	1.181 [0.992,1.405]	1.128 [0.608,2.092]	1.174 [0.978,1.409]
Middle	1.788^***^ [1.495,2.138]	1.601 [0.926,2.769]	1.774^***^ [1.465,2.147]
Richer	2.718^***^ [2.217,3.333]	3.339^***^ [1.944,5.736]	2.452^***^ [1.954,3.077]
Richest	4.898^***^ [3.547,6.764]	5.309^***^ [2.860,9.856]	6.064^***^ [3.542,10.38]
**Has media access (No)**	1	1	1
Yes	1.061 [0.931,1.209]	1.066 [0.739,1.537]	1.081 [0.938,1.246]
**Religion (Islam)**	1	1	1
Other	1.057 [0.924,1.210]	1.040 [0.739,1.463]	1.057 [0.912,1.226]
**Ethnicity (Emakhuwa)**	1	1	1
Portugais	2.688^***^ [1.540,4.692]	1.636 [0.764,3.503]	4.556^**^ [1.753,11.84]
Xichangana	1.557^***^ [1.215,1.996]	0.931 [0.556,1.559]	1.894^***^ [1.414,2.535]
Cisena	2.111^***^ [1.686,2.644]	5.427^***^ [2.450,12.02]	1.941^***^ [1.519,2.481]
Elomwe	0.531^***^ [0.391,0.720]	0.246^***^ [0.115,0.524]	0.618^**^ [0.443,0.864]
Cindau	1.876^***^ [1.423,2.474]	0.933 [0.373,2.330]	2.073^***^ [1.548,2.775]
Xitswa	0.949 [0.708,1.272]	0.723 [0.316,1.652]	1.010 [0.736,1.385]
Other	1.440^***^ [1.215,1.708]	1.065 [0.693,1.638]	1.555^***^ [1.288,1.877]
**Parity (1–5)**	1	1	1
> 5	0.995 [0.830,1.192]	0.886 [0.562,1.395]	1.001 [0.820,1.223]
**Household head’s sex (Male)**	1	1	1
Female	1.101 [0.965,1.257]	0.761 [0.561,1.033]	1.184^*^ [1.022,1.371]
**Child wantedness (Wanted Then)**	1	1	1
Wanted No More	1.059 [0.881,1.273]	1.383 [0.947,2.020]	0.984 [0.795,1.219]
**Antenatal Visits (< 4)**	1	1	1
4 or more	1.340^***^ [1.188,1.512]	1.655^***^ [1.235,2.218]	1.265^***^ [1.108,1.445]
Pseudo *R*^2^	0.184	0.190	0.199

Exponentiated coefficients; 95% confidence intervals in brackets

^*^ *p* < 0.05, ^**^ *p* < 0.01, ^***^ *p* < 0.001

**Table 3:** Predictors of using Caesarean Section Services in Mozambique

	Pooled	Urban	Rural
**Age (15–19)**	1	1	1
20–24	0.662^*^ [0.441,0.993]	0.546^*^ [0.325,0.917]	0.924 [0.474,1.800]
25–29	0.739 [0.491,1.110]	0.592^*^ [0.352,0.995]	1.079 [0.549,2.123]
30–34	0.755 [0.485,1.175]	0.680 [0.389,1.190]	0.877 [0.412,1.868]
35–39	1.044 [0.648,1.681]	0.854 [0.465,1.569]	1.504 [0.681,3.320]
40–44	1.463 [0.794,2.695]	1.282 [0.594,2.770]	1.717 [0.612,4.818]
45–49	1.366 [0.519,3.596]	0.858 [0.168,4.387]	2.057 [0.575,7.356]
**Residency (Urban)**	1		
Rural	0.546^***^ [0.396,0.752]		
**Education (No Education)**	1	1	1
Primary	1.327 [0.926,1.902]	0.969 [0.545,1.725]	1.541 [0.970,2.447]
Secondary	2.114^**^ [1.335,3.347]	1.461 [0.766,2.786]	2.979^**^ [1.425,6.226]
Higher	4.568^***^ [2.105,9.912]	3.269^*^ [1.327,8.050]	1.601 [0.926,2.769]
**Husband’s education (No Education)**	1	1	1
Primary	0.727 [0.528,1.003]	0.751 [0.466,1.212]	0.697 [0.448,1.085]
Secondary	0.779 [0.532,1.140]	0.777 [0.469,1.287]	0.825 [0.441,1.544]
Higher	1.280 [0.701,2.335]	1.430 [0.722,2.835]	1.020 [0.641,1.722]
**Employment (Not Working)**	1	1	1
Professional/Technical/Managerial	1.210 [0.921,1.588]	1.324 [0.966,1.814]	0.864 [0.472,1.581]
Agricultural - Self Employed	0.696^*^ [0.488,0.993]	0.675 [0.366,1.244]	0.654 [0.415,1.031]
**Wealth quintile (Poorest)**	1	1	1
Poorer	1.368 [0.749,2.498]	1.101 [0.909,1.334]	1.767 [0.903,3.457]
Middle	1.548 [0.873,2.745]	1.660 [0.579,4.757]	1.289 [0.635,2.617]
Richer	1.507 [0.850,2.670]	0.821 [0.289,2.330]	1.791 [0.875,3.665]
Richest	2.077^*^ [1.101,3.919]	1.336 [0.469,3.808]	2.277 [0.904,5.737]
**Has media access (No)**	1	1	1
Yes	0.926 [0.678,1.264]	0.921 [0.564,1.505]	0.956 [0.633,1.446]
**Religion (Islam)**	1	1	1
Other	0.812 [0.610,1.080]	0.993 [0.693,1.422]	0.637 [0.392,1.036]
**Ethnicity (Emakhuwa)**	1	1	1
Portugais	1.346 [0.815,2.224]	1.742 [0.910,3.336]	1.612 [0.570,4.561]
Xichangana	1.243 [0.775,1.993]	1.687 [0.892,3.190]	0.821 [0.377,1.788]
Cisena	1.325 [0.775,2.265]	1.759 [0.835,3.706]	1.085 [0.490,2.405]
Elomwe	0.487 [0.146,1.628]	0.563 [0.0712,4.457]	0.405 [0.0906,1.809]
Cindau	1.647 [0.908,2.987]	2.669^*^ [1.048,6.801]	1.254 [0.564,2.790]
Xitswa	1.817 [0.989,3.338]	2.443 [0.986,6.052]	1.508 [0.653,3.480]
Other	1.127 [0.731,1.738]	1.481 [0.793,2.768]	0.905 [0.490,1.674]
**Parity (1–5)**	1	1	1
> 5	0.627^*^ [0.407,0.964]	0.627 [0.339,1.157]	0.651 [0.346,1.224]
**Household head’s sex (Male)**	1	1	1
Female	1.122 [0.886,1.422]	1.101 [0.814,1.489]	1.109 [0.752,1.637]
**Child wantedness (Wanted Then)**	1	1	1
Wanted No More	0.930 [0.709,1.222]	0.821 [0.594,1.134]	1.379 [0.836,2.276]
Antenatal Visits (< 4)	1	1	1
4 or more	1.393^*^ [1.077,1.800]	1.270 [0.910,1.771]	1.570^*^ [1.042,2.365]
Pseudo *R*^2^	0.181	0.173	0.160

Exponentiated coefficients; 95% confidence intervals in brackets

^*^ *p* < 0.05, ^**^ *p* < 0.01, ^***^ *p* < 0.001

Similar results were observed for husband’s education as well, however the odds were significant only among rural women and for secondary [OR = 1.762, 95%CI = 1.328,2.338] and higher education only [OR = 1.236, 95%CI = 1.031,1.481]. Women from the middle [OR = 1.788, 95%CI = 1.495,2.138], richer [OR = 2.718, 95%CI = 2.217,3.333] and richest [OR = 4.898, 95%CI = 3.547,6.764] wealth quintile households had significantly higher odds of using facility delivery compared with those in the lowest quintile. Women of Portugais [OR = 2.688, 95%CI = 1.540,4.692], Cindau [OR = 1.876, 95%CI = 1.423,2.474] and Xichangana [OR = 1.557,1.215,1.996] ethnicity had relatively higher odds of using facility delivery services than others. Rural women in the female headed households had higher odds of using facility delivery services [OR = 1.184, 95%CI = 1.022,1.371]. Using adequate ANC visits was a significant predictor of facility delivery services both in urban [OR = 1.655, 95%CI = 1.235,2.218] and rural [OR = 1.265, 95%CI = 1.108,1.445] areas.

Regarding the use of c-section services, the most notable predictors were similar to that of facility delivery: rural residence, higher education, higher wealth, and adequate ANC visits. Having higher education showed positive association with the use of c-section among urban [OR = 3.269, 95%CI = 1.327,8.050] and rural residents with secondary education [OR = 2.979,1.425,6.226]. Cindau women in the urban areas [OR = 2.669, 95%CI = 1.048,6.801] had higher odds of using c-section. Among rural women, using adequate ANC visits was a significant predictor of choosing c-section delivery [OR = 1.570, 95%CI = 1.042,2.365].

## Discussion

Using the data from Mozambique Demographic and Health Survey, findings from this study revealed that little less than one-third of the women in Mozambique were not using health facility delivery services. In the region-specific analysis, the disparity was more evident among rural women, with the prevalence being two-fifth compared with about 90% among urban women. Urban-rural disparity in the use of maternal and reproductive services is common in Sub-Saharan African countries [24–28]. The growing urban-rural inequality in terms of using lifesaving services poses a major challenge for meeting the goals of reducing maternal and child mortality and morbidity [25]. Stark inequality was observed in the use of c-section services as well, with the prevalence being 10.2% in the urban and 2.9% in the rural areas. While prevalence of c-sections in urban areas aligned with the World Health Organization recommended cut-off of 10–15%, that of the rural areas was remarkably low [29]. Better availability of the medical infrastructure and awareness about the use of c-section services have spurred its global prevalence (~ 21%), which is far higher than the African average of about 5% [30]. Lower use of C-section services can stem from various causes such as affordability and awareness; these should be investigated and addressed to reduce inequality.

There were sociodemographic disparities in the utilization of facility delivery and c-section services. Women with higher education were significantly more likely to use facility delivery services compared with those who had no education. The positive effect of education on health service seeking behaviour can function in two ways. Firstly, educated women are more likely to be aware of the danger signs of pregnancy and the risk factors of pregnancy complications; hence, they are more likely to avail themselves of the services [31, 32]. Secondly, educated women are expected to be more financially empowered and have better decision-making autonomy [33]. These factors can enable them to make better use of the services which would otherwise be unaffordable or inaccessible to them. Furthermore, the findings revealed that women living in households with higher wealth status had higher odds of using both facility delivery and c-section services. Financial well-being is a predictor of general health status as well as positive healthcare-seeking behaviour. Therefore, removing financial constraints in accessing child-birth services should be considered an important priority in Mozambique.

Women’s ethnic background was an important predictor of using facility delivery services as well. The racial/ethnic differences in maternal health service use and health outcomes have been a subject of growing interest in many countries [34–37]. The underlying factors behind this difference generally consist of lower socioeconomic status and perception of health and healthcare [29, 38]. In some cultures, delivering at home may be considered normal or more culturally appropriate, thereby making the practice more common even when professional birthing services are available [38]. Sociocultural factors that shape healthcare-seeking behaviour are inherently more challenging to address and require special strategies such as replacing the existing norms and beliefs with the new ones through community education (role play) programs [39, 40]. It is important to design context-specific and locally-relevant interventions that increase uptake, avoiding the appearance of being culturally invasive [41].

Lastly, we found an association between the use of ANC visits and professional birthing services. ANC programs are generally educational and serve as the preparatory stage for safe delivery and successful termination of a pregnancy. Women who contact care-providers during the ANC stage are more likely to learn about and plan the place of delivery [42]. Promoting the use of ANC services appears to be an important driver for scaling up facility delivery programs.

This study provided relevant insights regarding the prevalence and use of facility delivery and c-section services use in a nationally-representative sample in Mozambique. Findings contribute to an existing gap in the literature and generate potential areas of investigation for future research. Significant differences were found in the use of facility delivery services among certain ethnicities. While these findings provide a general idea regarding the nature of ethnic inequality, it did not allow the investigation of underlying mechanisms contributing to the trend. Further qualitative studies should be carried out to explore the sources of disparity and potential areas of intervention.

Several limitations should be noted for this study. The data were cross-sectional and hence no causality can be inferred from the associations. Authors used a secondary database and, therefore, have no influence over the selection and measurement of the variables. As the data were self-reported, the chances of recall and reporting bias cannot be ignored. The factors that influence healthcare service utilization are diverse and multifaceted, but the choice of the explanatory factors was limited to what existed in the Mozambique 2011 DHS survey. Factors such as geographical distance, transportation facilities, quality of services in local healthcare settings, availability of female care provider have also been found to be important in primary studies; we were not able to adjust for these factors in the current analysis. The use of C-section services is also a complex outcome that can be driven by various personal and medical factors; no such data were collected in the Mozambique DHS. The survey was conducted in 2011, and therefore may not represent the current scenario. Lastly, due to the nature of the survey and analyses, causality cannot be inferred for the relationship between the outcomes (place of delivery and use of c-section services) and associated explanatory factors.

## Conclusion

This was a secondary analysis of the 2011 Mozambique Demographic and Health Survey data on the use of professional childbirth services among community dwelling women. The findings indicate that approximately 30% of the women were not using health facility delivery services, with the difference denoting a considerable urban-rural gap. Significant differences were also observed for women’s education, household wealth, and ethnic background.

## Data Availability

Data for this study were sourced from Demographic and Health surveys (DHS) and available here: http://dhsprogram.com/data/available-datasets.cfm.

## Abbreviations

ANC: Antenatal Care
CS: C-Section
DHS: Demographic and Health Surveys
MCM: Maternal and child mortality
TBAs: Traditional Birth Attendants
SBAs: Skilled birth attendants
WHO: World Health Organization
